# Metabolomic approach to key metabolites characterizing postmortem aged loin muscle of Japanese Black (Wagyu) cattle

**DOI:** 10.5713/ajas.18.0648

**Published:** 2019-01-04

**Authors:** Susumu Muroya, Mika Oe, Koichi Ojima, Akira Watanabe

**Affiliations:** 1 Muscle Biology Research Unit, Animal Products Research Division, NARO Institute of Livestock and Grassland Science, Tsukuba, Ibaraki 305-0901, Japan; 2 NARO Tohoku Agricultural Research Center, Morioka, Iwate 020-0198, Japan

**Keywords:** Beef, Capillary Electrophoresis Time-of-flight Mass Spectrometry (CE-TOFMS), Japanese Black, Metabolomics, Postmortem Aging, Wagyu

## Abstract

**Objective:**

Meat quality attributes in postmortem muscle tissues depend on skeletal muscle metabolites. The objective of this study was to determine the key metabolic compounds and pathways that are associated with postmortem aging and beef quality in Japanese Black cattle (JB; a Japanese Wagyu breed with highly marbled beef).

**Methods:**

Lean portions of *Longissimus thoracis* (LT: loin) muscle in 3 JB steers were collected at 0, 1, and 14 days after slaughter. The metabolomic profiles of the samples were analyzed by capillary electrophoresis time-of-flight mass spectrometry, followed by statistical and multivariate analyses with bioinformatics resources.

**Results:**

Among the total 171 annotated compounds, the contents of gluconic acid, gluconolactone, spermidine, and the nutritionally vital substances (choline, thiamine, and nicotinamide) were elevated through the course of postmortem aging. The contents of glycolytic compounds increased along with the generation of lactic acid as the beef aging progressed. Moreover, the contents of several dipeptides and 16 amino acids, including glutamate and aromatic and branched-chain amino acids, were elevated over time, suggesting postmortem protein degradation in the muscle. Adenosine triphosphate degradation also progressed, resulting in the generation of inosine, xanthine, and hypoxanthine via the temporal increase in inosine 5′-monophosphate. Cysteine-glutathione disulfide, thiamine, and choline increased over time during the postmortem muscle aging. In the Kyoto encyclopedia of genes and genomes database, a bioinformatics resource, the postmortem metabolomic changes in LT muscle were characterized as pathways mainly related to protein digestion, glycolysis, citric acid cycle, pyruvate metabolism, pentose phosphate metabolism, nicotinamide metabolism, glycerophospholipid metabolism, purine metabolism, and glutathione metabolism.

**Conclusion:**

The compounds accumulating in aged beef were shown to be nutritionally vital substances and flavor components, as well as potential useful biomarkers of aging. The present metabolomic data during postmortem aging contribute to further understanding of the beef quality of JB and other breeds.

## INTRODUCTION

Skeletal muscle metabolites such as amino acids, peptides, nucleotide-related products, fatty acids, and sugars contribute to meat quality as flavor components. Postmortem protein degradation generates free amino acids and peptides in the muscles of cattle [1]. These compounds contribute to the development of meat flavor in beef by Maillard reaction in cooked foods, one of the most important molecular events for flavor formation, which occurs primarily between amino acids and reducing monosaccharides [2]. In pork, inosine 5′-monophosphate (IMP), ribose, and glucose increase “meaty” aroma, while IMP, ribose, and glucose 6-phosphate increase “roasted” aroma [3].

Meanwhile, nucleotide triphosphates such as adenosine triphosphate (ATP) and guanosine triphosphate (GTP) are broken down during postmortem meat aging, generating taste-associated products including IMP and guanosine monophosphate (GMP) [4]. Moreover, lactic acid accumulation caused by glycolysis results in a decline in muscle pH [5]. This indirectly affects water-holding capacity, color, and tenderness of meat through protein denaturation and protease inactivation. Thus, most of the low-molecular-weight water-soluble metabolites present in skeletal muscle are generated, and some of these are further converted to meat flavor components during meat aging and cooking processes. Despite the importance of these water-soluble compounds in meat, metabolomic changes by which these metabolites are generated in beef have been poorly explored.

A comprehensive understanding of the changes that occur in postmortem muscle metabolites could provide key information on how to control the generation of key compounds for development of meat quality. Metabolomic approaches to beef cattle have been applied to exploration of metabolites related to sensory characteristics and beef flavor [6,7], differences in cattle feeding systems [8], and the storage conditions of minced beef [9] using liquid chromatography-mass spectrometry (LC-MS), gas chromatography (GC)-MS, and headspace solid phase microextraction-GC/MS methodologies. A metabolomic study on postmortem beef aging, using reverse-phase high-performance (HP) LC-MS-based metabolomics, has revealed involvement of metabolites including acyl carnitines and amino acids in beef color stability and lipid oxidation during postmortem aging [10]. The HPLC-MS-based technique has the advantage of detecting semipolar metabolites but not polar metabolites such as ATP and organic acids, due to the higher retention on less charged compounds rather than charged compounds. Another metabolomic study of beef aging using nuclear magnetic resonance (NMR) spectroscopy obtained a total of 25 annotated metabolites in Japanese Black (JB; a Japanese Wagyu breed) cattle, and demonstrated that NMR-based metabolomics can evaluate multiple parameters related to the beef-quality attributes of this breed [11].

In pork analysis, capillary electrophoresis time-of-flight MS (CE-TOFMS) was previously employed for the analysis of changes in the metabolomic profiles of porcine muscles during postmortem aging, revealing the relevant pathways that generate metabolites associated with pork quality, including the postmortem energy metabolism and protein degradation pathways [12]. The results demonstrated the efficacy of CE-TOFMS technique for such studies based on its broader coverage and the identification of water-soluble, charged metabolites.

JB cattle are well known for their genetically superior intramuscular fat depot, which makes beef tender and gives JB beef the high marbling score that is so prized. In the present study, to explore the key metabolites and pathways in postmortem beef aging, changes in profiles of water-soluble metabolites during postmortem aging of JB beef were comprehensively analyzed for the first time. The CE-TOFMS technique was used for the analysis of water-soluble charged metabolites including free amino acids, organic acids, and short-chain peptides in the lean portion of JB cattle *Longissimus thoracis* (LT) muscle.

## MATERIALS AND METHODS

### Animals and muscle samples

The animals were cared for as outlined in the Guide for the Care and Use of Experimental Animals (Animal Care Committee of the NARO Institute of Livestock and Grassland Science [NILGS]), and this committee approved the study (approval number 1611C010). All efforts were made to minimize suffering of the animals. A portion of the LT muscles at the 8th to 9th thoracic vertebrae was dissected from three JB steers aged 28 months (632 to 739 kg) at 30 min after slaughter at the slaughter house of the NILGS. After intramuscular fat was carefully removed from the muscle samples, several pieces of small lean-muscle were picked up from four or five locations in the core part (approx. 2-cm diameter) of each LT muscle at time 0 postmortem. To minimize the deterioration of metabolites, mincing of the muscle samples was avoided. After overnight hanging of the carcasses at 2°C, the remaining LT muscles were excised out, and lean muscle samples were taken from the LT muscles at 24 h (1 d); then, after continuous storage of the LT muscles at 2°C, further muscle pieces were picked up at 336 h (14 d) postmortem. The mean values of the crude fat, protein, and moisture contents of the LT muscle block samples were 365.3 mg/g (36.5%), 141.7 mg/g (14.2%), and 476.7 mg/g (47.7%), respectively. After collection from the carcasses, intramuscular fat was manually removed from the muscle tissues. All the subsequent small pieces of lean muscle were collected and frozen in liquid nitrogen and thereafter stored at −80°C until use. Muscle pH was measured according to the method described previously [12].

### Sample preparation for CE-TOFMS

The frozen muscle pieces (46.3 to 90.0 mg) were immediately plunged into a solution (50% acetonitrile, 10 μM Internal Standard Solution 1 [Human Metabolome Technologies, Tsuruoka, Japan]) at 0°C and homogenized twice at 1,500 rpm for 120 s. The samples were then centrifuged at 2,300×*g* for 5 min at 4°C. The upper layer solution was centrifugally filtered through a Millipore 5-kDa cutoff membrane. The filtrate was lyophilized, suspended in Milli-Q water and analyzed by CE-TOFMS.

### Instrumentation and conditions of CE-TOFMS

CE-TOFMS was carried out using an Agilent CE Capillary Electrophoresis System equipped with an Agilent 6210 Time-of-Flight mass spectrometer, Agilent 1100 isocratic HPLC pump, Agilent G1603A CE-MS adapter kit, and Agilent G1607A CE-ESI-MS sprayer kit (Agilent Technologies, Waldbronn, Germany). The analytic conditions were the same as those used in a previous study [12]. The spectrometer was scanned from *m/z* 50 to 1,000.

### Data analysis of CE-TOFMS results

Raw data obtained by CE-TOFMS were processed with MasterHands, as in our previous study [12]. Among all the detected compounds, the compounds annotated in the Human Metabolome Database (ver. 4.0, http://www.hmdb.ca/) or the Kyoto encyclopedia of genes and genomes database (KEGG; http://www.genome.jp/kegg/), a bioinformatics resource, were further analyzed. The relative contents of the annotated compounds over time were determined by comparing the peaks of compounds with the same MS properties in the analysis. To compare the relative contents of the compounds between time points, peak areas were normalized by those of the internal standards (methionine sulfone for cations, (+)-camphor-10-sulfonic acid for anions) and by sample weight. The resultant relative area values but not the absolute contents were further normalized by the content of methylhistidine (MH; sum of 1 MH and 3 MH, 3 MH is a skeletal muscle-specific metabolite [13]) which is not affected by postmortem aging time in pork (unpublished data). The contents of major metabolites such as glycolytic products, amino acids, and ATP degradation products were determined using commercially available standards. The abundance of each compound used for comparative analysis was set as 0 when the content of the compound was under detection. The annotated compounds were categorized using the KEGG database, to extract the terms that represent molecular metabolic events associated with the categorized compounds. File conversion of raw MS data, peak picking, reduction of noise, and alignment of data for multiple samples were conducted as previously described [12].

### Statistical analyses

The normalized relative content values were used for the data analyses. Cluster 3.0 (http://bonsai.hgc.jp/~mdehoon/software/cluster/software.htm) combined with Java TreeView software (https://sourceforge.net/projects/jtreeview/) was used for hierarchical cluster analysis (HCA). The principal component analysis (PCA), analysis of variance (ANOVA), and multiple comparison test were conducted using SAS Excel Add-In 6.1 for Microsoft Office (https://www.sas.com/ja_jp/home.html). For PCA, all the relative values were transformed into Z-scores, to calculate the principal component (PC) scores and loading values.

To assess the significance of differences between the time points for each compound in multiple comparisons, statistical analysis was performed using R 3.5.1 (http://cran.r-project.org), using an R package for the analysis of significant gene expression profile differences over time (maSigPro release 3.7; https://www.bioconductor.org/packages/release/bioc/html/maSigPro.html). An adjusted p-value (maSigPro *P*) <0.050, corresponding to a false discovery rate (FDR) <0.10 and p-values <0.05 were considered statistically significant.

Data were also statistically analyzed using one-way ANOVA for each compound. The model included the fixed effects of postmortem storage time and a random effect of the individual animal, considering the factor of storage time to be within-subject. Data were considered significantly different if p<0.05 in a post hoc multiple test using Fisher’s least significant difference test (LSD) after ANOVA. The pH value was also analyzed by ANOVA, followed by a post hoc multiple test with the LSD.

## RESULTS

### Quantitative analyses of postmortem changes in metabolites of LT muscle in JB steers by CE-TOFMS

To clarify the metabolic characteristics and factors responsible for metabolic events in the LT muscle at each time point, metabolomic analyses using CE-TOFMS were conducted. In total, 197 compounds were detected in the beef samples, among which 171 compounds (117 cations and 54 anions) were annotated. Other peaks corresponding to a total of 26 compounds were also detected but could not be determined due to a lack of structural information. Among the 171 annotated metabolites, a total of 70 compounds were absolutely quantified using standard compounds (Table 1). Of the 70 compounds, the contents of 31 increased over time, while the contents of 14 decreased (p<0.05); the rest showed no difference between 0 d and 14 d, with or without change at 1 d. Although there were 110 metabolites to be potentially quantified by the present methodology, 40 of those were not detected (data not shown). Charged metabolites associated with glycolytic metabolism and the citric acid cycle (i.e., the tricarboxylic acid cycle) are not easily detected by LC-MS- or GC-MS-based methods [10], but they were successfully quantified using the present approach.

**Figure 1:**
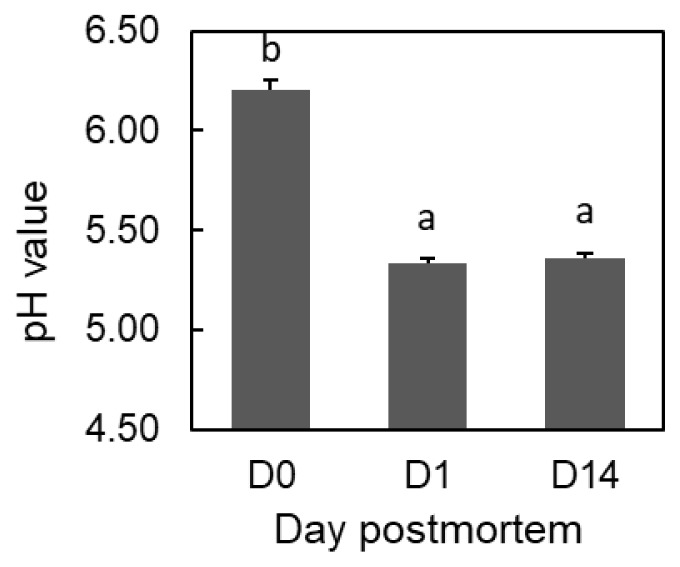
Postmortem pH decline in *Longissimus thoracis* muscle of Japanese Black steers. Different letters (a, b) indicate significant differences among time points (p<0.001). Error bars indicate standard error (n = 3).

According to the results regarding the glycolytic pathway, the lactic acid content increased significantly within the first 24 hrs (p = 0.02). In contrast, the fructose 1,6-bisphosphate (F-1,6-dP) content decreased over the first 24 hrs, indicating the progress of glycolysis in the LT muscle in the early postmortem period. On the other hand, ATP degradation, temporary IMP accumulation at 1 d, and the subsequent accumulation of hypoxanthine and inosine progressed in a coordinated manner during aging. Concomitantly, the muscle pH, starting at 6.20 at 1 h postmortem, declined to 5.33 within the first 24 hrs, and thereafter remained at the ultimate pH value (5.36) until 14 d postmortem (Figure 1). The contents of 15 protein constituent amino acids significantly increased in the postmortem period (Table 1; p<0.05).

### Characterization of aging beef samples by HCA and PCA

**Figure 2:**
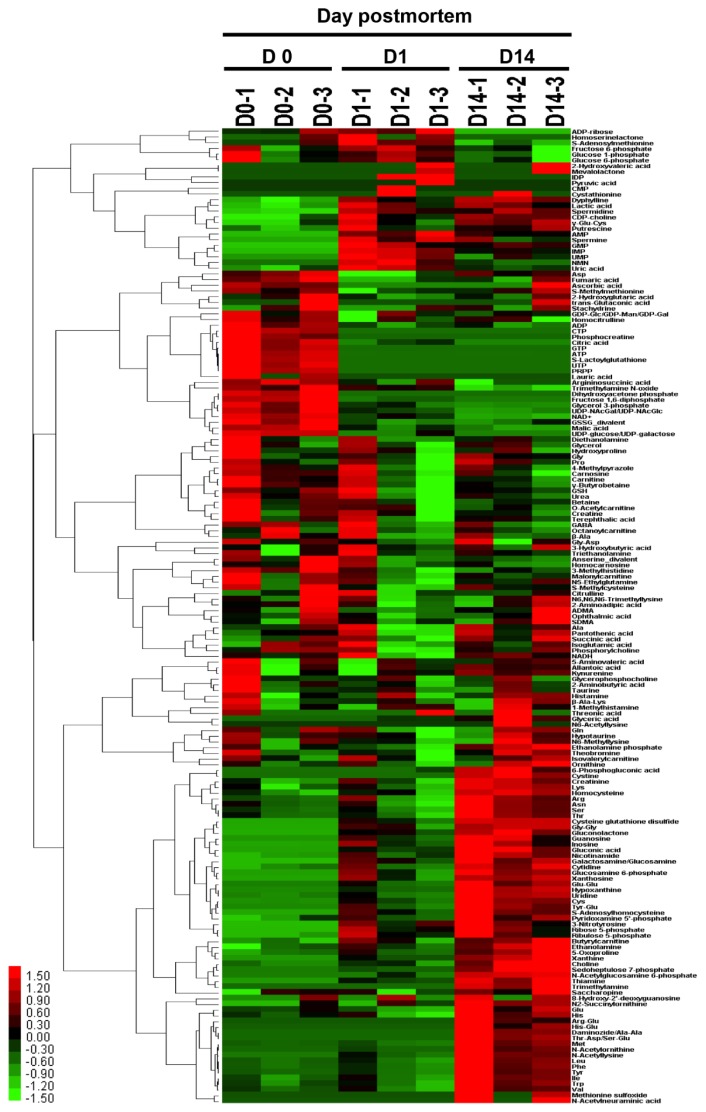
Heatmap result of hierarchical clustering analysis of metabolomic changes during postmortem aging in *Longissimus thoracis* muscle of Japanese Black steers. Three cattle samples were allocated to each group at a specific time point (day 0: D0, day 1: D1, day 14: D14). The row displays the metabolite and the column represents the sample. Metabolites with relatively low contents are displayed in green, while metabolites with relatively high contents are displayed in red. The brightness of each color corresponds to the magnitude of the difference when compared with the average value.

To characterize the muscle samples at each time point using the metabolomic profiles, the resultant profiles were applied to HCA and PCA analyses using all the metabolites detected and annotated. Before the comparison, it was confirmed that the MH content was not significantly affected by aging time (p>0.05 in ANOVA), which indicates its usefulness as an internal standard of postmortem lean muscle content (data not shown). The heatmap resulting from HCA clearly showed that the metabolomic composition of the beef water-soluble compounds was separated into several categories according to the pattern of changes over time (Figure 2). In other words, the samples were grouped by the pattern of postmortem changes in water-soluble compounds detected in the lean portion of JB beef.

**Figure 3:**
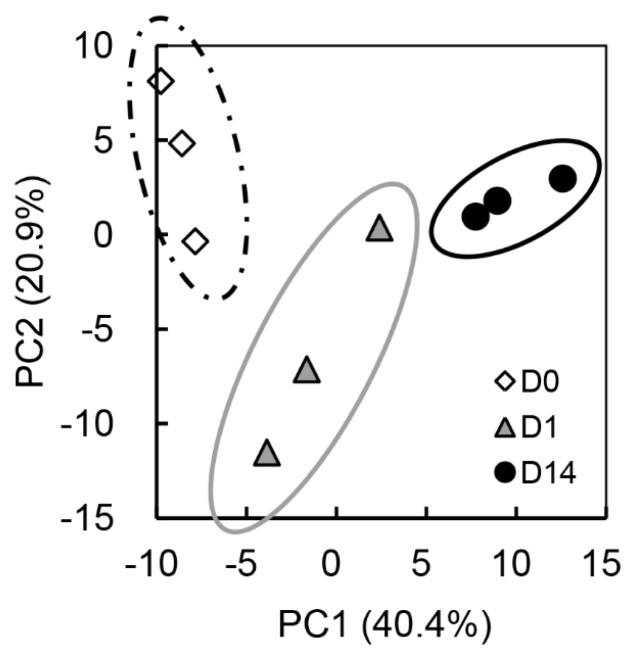
Principal component analysis of *Longissimus thoracis* muscle of Japanese Black steers by metabolomic changes during postmortem aging. The plots of open square (D0), gray triangle (D1), and closed circle (D14) indicate LT samples taken at 0, 24 h (1 d), and 168 h (14 d) postmortem, respectively.

The PCA results also showed that the metabolomic profile of the beef could be grouped according to the first two PCs, PC1, and PC2 (Figure 3). The cumulative proportions of PC1, PC2, and PC3 were 40.4%, 20.9%, and 11.9%, respectively. The plot patterns of the beef samples at D0, D1, and D14 revealed a clear association of PC1 with postmortem muscle aging. In particular, the D0 and D14 samples were segregated from each other by PC1, with the intermediate D1 samples as a transient state.

The loading scores of cysteine-glutathione disulfide (Cys-GTdS), thiamine, nicotinamide, gluconic acid, Cys, gluconic acid, and hypoxanthine were highly positive for PC1 (Table 2). The D0 and D14 samples were plotted in the negative and positive areas of PC1, respectively, indicating that the JB beef samples at day 14 postmortem were characterized by an abundance of the above-mentioned compounds, such as thiamine and Cys-GTdS. In addition, PC1 was negatively attributed to oxidized form of nicotinamide adenine dinucleotide (NAD+), F-1,6-dP, glycerol 3-phosphate, citric acid, malic acid, cytidine triphosphate, GTP, and uridine diphosphate (UDP)-linked compounds, indicating that these compounds were abundant in the muscle at slaughter but later decreased. On the other hand, the beef samples at D1 were grouped and plotted in a negative area of PC2 compared to those at D0 and D14 (Figure 3). The compounds including Gly, urea, betaine, glutathione, and reduced form of nicotinamide adenine dinucleotide (NADH) showed positive loading scores, while compounds such as GMP, uric acid, spermine, uridine monophosphate (UMP), IMP, adenosine monophosphate (AMP), and inosine diphosphate showed negative loading scores for PC2 (Table 2).

### Highlighted metabolic pathways and compounds in postmortem LT muscle of JB steers

In addition to absolute quantification of the major metabolites, the relative contents of the annotated compounds were also determined to elucidate the biological events associated with the annotated compounds including those whose standard compound was not available. By evaluating the significance of postmortem changes in the metabolite contents in the global JB muscle metabolome, it would be possible to extract not only the relevant metabolic pathways that were activated in the beef samples during postmortem aging, but also significant metabolites that characterize the aged JB beef. To this end, maSigPro software (a statistical R-based package) was employed to test multiple comparisons against changes in the relative contents of the annotated metabolites of the beef, including the absolutely quantified compounds. Consequently, a total of 89 compounds showing significant increases or decreases during postmortem aging (adjusted p<0.05, FDR<0.10) were extracted, of which the top 50 compounds are shown in Supplementary Table S1. Of those, not only 16 amino acids, but also the contents of 6 dipeptides also increased over the duration (Table 3). In addition to compounds related to the glycolytic pathway (dihydroxyacetone phosphate [DHAP], F-1,6-dP, lactic acid) and purine metabolism (AMP, ATP, GMP, hypoxanthine, IP, etc.), other compounds, including 6-phosphogluconic acid (6PG), sedoheptulose 7-phosphate (S7P), citric acid, fumaric acid, and NAD+, showed changes in their contents in the postmortem period.

Using the KEGG database, all the significantly changed compounds (p<0.05) were assigned to functional pathways representing postmortem aging of the LT muscle. We extracted the pathways in which at least three compounds changed significantly (p<0.05); these included the citric acid cycle, glycerophospholipid metabolism, cysteine metabolism, protein digestion and absorption, nicotinate and nicotinamide metabolism, purine metabolism, pentose phosphate metabolism, and polyamine metabolism pathways (Table 3). It is therefore suggested that these metabolic pathways are prominent in the lean portion of JB beef during postmortem aging.

**Figure 4:**
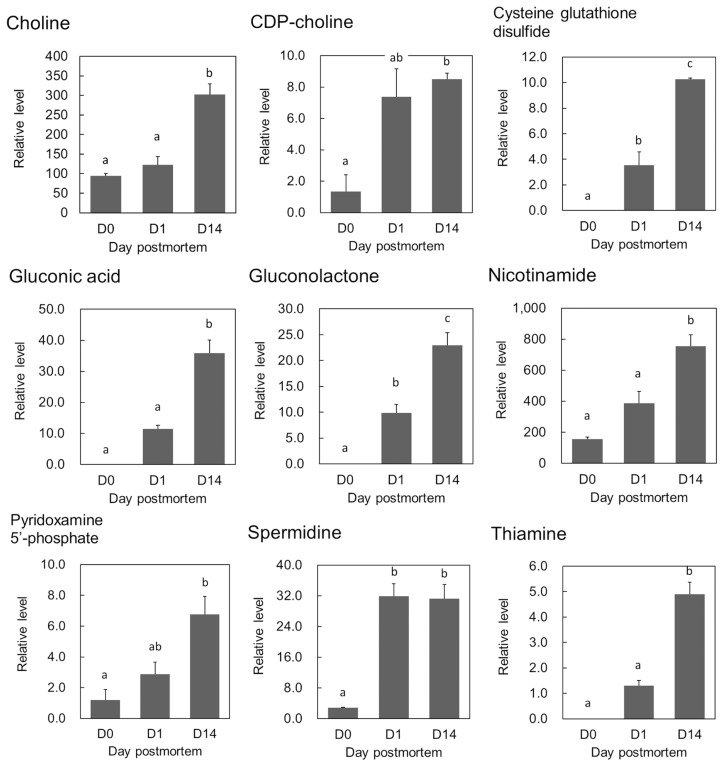
Postmortem changes in relative contents of metabolites in *Longissimus thoracis* muscle of Japanese Black steers. The relative content contents (vertical axis) were normalized using a method described in Materials and Methods. The horizontal axis indicates postmortem time (day 0: D0, day 1: D1, day 14: D14). Error bars indicate standard error (n = 3). Different letters (a, b, c) indicate significant differences among time points (p<0.05) in post hoc least significant difference test after analysis of variance (p = 0.05 for pyridoxamine 5′-phosphate; p = 0.07 for CDP-choline; p<0.05 for the other compounds).

In addition to the above-mentioned metabolites related to beef quality, changes over time were observed in the contents of key metabolites related to nutrition and beef aging: the relative contents of gluconic acid, gluconolactone, Cys-GTdS and coenzyme precursors including pyridoxamine 5′-phosphate (PMP) and thiamine significantly increased during postmortem aging of the beef (adjusted p<0.05, FDR<0.10; Figure 4). Moreover, the relative contents of 6PG, 5-oxoproline, and N-acetyllysine increased, while that of phosphoribosyl pyrophosphate (PRPP) decreased during beef aging (adjusted p<0.05, FDR<0.10; Supplementary Table S1).

## DISCUSSION

### Major metabolic pathways observed during postmortem aging of the LT muscle in JB steers

In the present study, the contents of the metabolites in the lean LT muscle at different stages of postmortem aging were determined. Comparison of the contents of the annotated compounds among the postmortem time points by HCA, PCA, and the multiple comparison test revealed changes in numerous metabolites, including glycolytic products, energy metabolism-related compounds, purine and pyrimidine-related compounds, and amino acids. The pathways related to the glycolysis, purine metabolism, protein degradation, pentose pathway, and nicotinamide metabolism in the KEGG database were extracted as the molecular biological events associated with the significantly changed compounds (p< 0.05) during the postmortem aging of the LT muscle.

To focus on the changes in the lean muscle metabolites, the potential contaminating influence of intramuscular fat was excluded by taking only the lean muscle portion as the samples, which was followed by normalization with MH, the content of which did not differ among the aging time points. As a result, the predicted postmortem biochemical events observed during pork aging [12], such as increases in amino acids and ATP degradation products, were observed (Table 3). This means that the MH-based CE-TOFMS analysis of the metabolite contents in lean muscle portion of Wagyu cattle was not affected by variation of the intramuscular fat content of the cattle. The observations confirmed that the present muscle samples underwent a typical postmortem aging process [5], and that postmortem metabolic changes in bovine LT muscle are very similar to those in porcine muscle [12].

### Protein digestion and absorption

During postmortem aging of the LT muscle, 16 free amino acids increased (Table 3), which indicates that the muscle proteins were degraded as demonstrated in the progress of porcine [12,14] and bovine muscle proteolysis [15–17]. This protein degradation process is reemphasized by the elevated contents of several dipeptides such as Glu-Glu (p<0.05), a dipeptide sequence frequently present in troponin-T [15,18] that is easily degraded in postmortem bovine muscles [16,17]. The accumulation of amino acids during postmortem aging was also observed in beef of Angus×Simmental crossbred steers [10]. Taken together, these results suggest that postmortem proteolysis generated free amino acids via the breakdown of muscle proteins to dipeptides after 1 d. It is also likely that endoproteinases such as calpains and aminopeptidases [14,19] played principal roles in the generation of most of the amino acids present after 1 d postmortem. The Asp and Glu thus generated might participate in a series of reactions to provide substrates for the citric acid cycle as glucogenic amino acids in the postmortem muscle [20].

The progress of muscle proteolysis is expected to contribute to postmortem tenderization in the JB loin beef, as shown in past beef studies [21]. The generation of amino acids and dipeptides also contributes to the improvement of beef flavor. The addition of monosodium glutamate to beef soup enhanced its “salty” or “potato” flavors in a consumer sensory examination [22]. Moreover, some dipeptides such as Glu-Glu are recognized to enhance umami taste of 0.02% IMP [23], suggesting that these proteolytic products are able to improve taste and flavor of beef.

### Citric acid cycle and pyruvate metabolism

Compounds involved in the citric acid cycle and pyruvate metabolism were decreased in the postmortem aging period (adjusted p<0.05, FDR<0.10) (Table 3). Lactic acid is a final product of glycolysis, emphasizing the progress of glycolysis in the postmortem LT muscle. The relative contents of citric acid, fumaric acid, and malic acid in the citric acid cycle decreased (adjusted p<0.05, FDR<0.10), suggesting that these compounds were supplied as a source of carbon chains for maintenance of homeostasis. DHAP may have been generated by the degradation of F-1,6-dP, which would account for the increase in DHAP in this study. DHAP is an important metabolite as a source of triglyceride synthesis and the pentose phosphate pathway [20], and thus the change in DHAP content might influence multiple metabolic pathways.

### Pentose phosphate pathway, nicotinate and nicotinamide metabolism

It is possible that the decrease in DHAP content influences changes in the contents of pentose phosphate pathway metabolites such as S7P and ribulose 5-phosphate (Ru5P). In fact, the S7P and Ru5P contents significantly increased (adjusted p<0.05, FDR<0.10) (Table 3). On the other hand, NADPH was not detected in this study. DHAP is made by coupling reactions with the pentose phosphate pathway as an energy source for synthesis of fatty acid and cholesterol [20]. Although the reason why neither NADPH nor NADP+ was detected remains unclear, previously those compounds were successfully detected by CE-TOFMS in pork [12]. There might be a specific difference between beef and pork in the role of NADPH/NADP+ in energy metabolism. Another energy-supplying compound, NADH, was detected, but its content did not change. In contrast, the NAD+ content decreased, possibly due to degradation to nicotinamide, the content of which increased during the aging period. The changes in the ratio of NADH/NAD+ were very similar to those in pork [12].

### Glycerophospholipid metabolism

This is the first study to report increases in choline and CDP-choline during postmortem aging of beef. Both choline and CDP-choline are nutritionally essential components as methyl-group donors in food materials, similarly to eggs [24]. These metabolites are categorized as part of glycerophospholipid metabolism, and such changes could reflect a shift of the metabolism toward a supply of energy source compounds in glycolytic metabolism where the final product is ATP. It is likely that the increases in choline and CDP-choline were linked with a metabolic shift toward G3P generation via DHAP by degradation of phosphatidyl choline, a plasma membrane component. Likewise, an increase in ethanolamine (data not shown) and its phosphate was also observed, possibly due to degradation of phosphatidyl ethanolamine, another component of the plasma membrane.

### Purine metabolism

A series of changes in metabolites associated with purine metabolism were also observed. This indicates that there was a dynamic change in the nucleotide metabolite profile over time during postmortem aging. Thus, ATP was degraded into xanthine or hypoxanthine via IMP or inosine generation, and GTP was degraded into GMP and guanosine in parallel during the aging process, resulting in increased contents of uric acid and PRPP. The 5′-ribonucleotides, AMP, IMP, and GMP, are all important in meat flavor perception, as they have umami taste characteristics [25] as well as an enhancing effect on umami taste. IMP and hypoxanthine are especially associated with meaty and bitter flavors in pork, respectively [26]. Thus, the catabolic reactions of purine metabolism contribute to the accumulation of taste-related compounds such as IMP in beef after appropriate aging periods.

### Characteristic compounds in postmortem aging of LT muscle in JB steers

During postmortem aging of beef in the present study, we clearly observed significant changes in metabolites that have the potential to influence human health. Some of these compounds, namely Cys-GTdS, nicotinamide, thiamine, and gluconic acid, have highly positive PC1 loading values, indicating that they are characteristic of postmortem aging of beef.

Gluconic acid and the precursor gluconolactone can promote growth of intestinal *Bifidobacterium*, since oral administration of gluconate increases the number of fecal *Bifidobacteria*, resulting in increased defecation frequency in humans [27]. The polyamines spermidine and putrescine were also detected in the present study. Changes in those polyamines during postmortem beef aging have not been reported previously. Spermidine also has a role of promoting microbiota growth in the intestine and thereby delaying senescence in mice [28]. Spermidine is a final compound in Gln degradation via ornithine and putrescine [20], which seemed to progress in postmortem LT muscle in the present study. Thiamine and PMP are well known as precursors of vitamins B2 and B6, respectively, which are essential nutrients for humans, as is nicotinamide [20]. The increases in the metabolites discussed above (i.e., choline, gluconate, polyamines, and vitamins) during the aging process may enhance the functional value of the beef.

In addition, according to the PCA loading analysis, thiamine is one of the most characteristic metabolites to show the extent of postmortem aging of the LT muscle, which has also been confirmed in porcine muscles [29,30]. Recently we hypothesized that thiamine may be generated by sequential dephosphorylation steps starting from thiamine triphosphate (ThTP) via thiamine diphosphate (ThDP) in the postmortem porcine muscles [30]. As thiamine is more absolvable than ThDP in intestinal epithelial cells [31], an increase in thiamine in aged beef may be beneficial for human health. Interestingly, neither ThTP nor ThDP was detected in the LT muscle during aging, even when using the same methodology as in the previous pork study. This difference might be due to the interspecies difference in metabolism, as suggested by the different contents of ThTP found in bovine and porcine muscles [29]. The mechanism of postmortem thiamine accumulation in bovine muscle requires further investigation.

Several other compounds have been shown to be characteristic of the postmortem aging of beef. PCA loading analysis showed higher contributions of Cys-GTdS (Figure 4) and hypoxanthine in the aged beef. In beef from Angus crossbred steers, the contents of acylcarnitines (butyryl-, pivaloyl-, hexanoyl-, octanoyl-, decanoyl-, and propionylcarnitines) were temporarily elevated during the aging period [10]. In the present study, butyryl-, malonyl-, isovaleryl-, and octanoylcarnitines were detected, but only butyrylcarnitine increased in the postmortem LT muscle (p<0.05, data not shown). The difference in detected compounds between the two studies might have been due to the different breeds of cattle studied, rather than the different detection methods used.

### Limitation

Several potential limitations should be mentioned. In the present study, we only used three steers, which is a minimal number for experiments. Moreover, there was a variation of body weight among the tested steers, and the impact of body weight on postmortem metabolite generation remains unknown. Nevertheless, our finding of significant changes in metabolite contents agreed with the results of past studies of postmortem meat aging, especially in regard to ATP degradation and the generation of amino acids and peptides. This suggests that the overall tendency of metabolomic changes observed in the three steers in this study would not contradict that observed in studies using larger numbers of animals, although further analyses are necessary to confirm those results.

### Conclusion

In the postmortem aging process in the LT muscle of JB steer, glycolysis, the citric acid cycle, the pentose phosphate pathway, protein digestion, amino acid generation, and purine metabolism were highlighted as the characteristic biochemical events. The accumulation of products in those pathways, such as IMP, R5P, and Glu, is expected to contribute to the improvement of the quality traits of aged beef. In addition, thiamine, choline, and Cys-GTdS could be useful as biomarkers of aged beef. Due to the accumulation of some of nutritional ingredients, the aging of beef may increase its value as a food and may contribute to human health.

## Supplementary Data



## Figures and Tables

**Table 1:** Changes in the contents of compounds during postmortem Japanese Black beef aging

Compound	D0		D1		D14		ANOVA p-value
		
	Mean (nmol/g)	SE	Mean (nmol/g)	SE	Mean (nmol/g)	SE	
3-Hydroxybutyric acid	136.6	22.8	211.3	30.7	198.0	29.0	0.069
6-Phosphogluconic acid	0	-a	19.1	0^a^	75.4	17.7^b^	0.021
ADP	53.5	2.1	35.2	9.9	31.0	4.5	0.119
Ala	2,295.2	380.1	2,427.6	246.5	2,930.5	241.8	0.283
AMP	0	-	99.1	39.8	47.7	3.5	0.092
Arg	334.5	35.7^a^	405.7	11.7^a^	641.4	44.3^b^	0.013
Asn	111.5	5.8^a^	114.6	5.6^a^	208.2	16.0^b^	0.008
Asp	44.1	9.3	13.1	13.1	39.3	3.5	NA
ATP	6,534.6	451.5^b^	21.1	7.0^a^	16.4	2.7^a^	<0.001
Betaine	1,712.7	69.7	1,611.4	214.4	1,560.7	84.1	0.689
Carnosine	12,381.8	1,028.1	1,2587.4	1,069.8	11,076.5	1,387.0	0.706
Choline	35.6	1.9^a^	58.4	13.3^a^	134.4	17.9^b^	0.006
Citric acid	113.7	9.4^b^	43.3	10.5^a^	0	-a	0.002
Citrulline	84.7	26.3	86.0	17.3	79.6	9.8	0.925
Creatine	21,093.9	1,605.9	23,799.2	3,311.2	20,804.1	504.8	0.610
Creatinine	479.6	10.7^a^	678.1	74.4^b^	1,019.7	29.5^c^	0.004
CTP	46.6	1.5^b^	0	-a	0	-a	<0.001
Cys	1.6	0.8^a^	23.6	6.9^a^	107.2	19.0^b^	0.005
Cytidine	2.4	0.7^a^	5.2	0.5^a^	12.0	1.4^b^	0.006
Dihydroxyacetone phosphate	801.1	113.7^b^	0	-a	0	-a	0.001
Fructose 1,6-diphosphate	168.8	33.2^b^	24.3	2.7^a^	0	-a	0.004
Fructose 6-phosphate	2,343.4	188.8	3,994.1	854.2	2,200.8	365.1	0.178
Fumaric acid	166.4	41.5	35.3	35.3	115.8	40.2	NA
GABA	19.4	2.9	21.0	1.8	18.4	2.1	0.804
Gln	3,613.6	530.1	3,228.4	177.8	4,126.7	707.3	0.278
Glu	312.4	68.0^a^	151.2	17.7^a^	776.0	165.1^b^	0.032
Gluconic acid	0	-	27.0	4.6	77.0	8.8	0.003
Glucose 1-phosphate	598.9	57.3	938.4	239.5	427.9	65.0	0.204
Glucose 6-phosphate	10,707.5	825.7	16,657.2	3,995.0	8,776.5	1,544.9	0.226
Glutathione (GSH)	696.7	77.9	711.5	56.7	707.2	7.4	0.961
Glutathione (GSSG)_divalent	91.5	9.7^b^	44.5	8.9^a^	45.1	9.5^a^	<0.001
Gly	1,083.4	15.7	1,121.1	164.7	1,272.8	62.6	0.537
Glycerol 3-phosphate	4,032.4	715.9^b^	1,087.3	493.4^a^	90.0	20.9^a^	0.018
GMP	0	-a	118.2	19.4^b^	78.1	6.9^b^	0.003
GTP	171.5	9.1^b^	0	-a	0	-a	<0.001
Guanosine	0	-a	4.7	1.1^a^	14.1	3.1^b^	0.007
His	152.1	19.0^a^	151.5	11.3^a^	256.4	23.8^b^	0.029
Hydroxyproline	45.2	3.6	47.5	8.5	44.0	7.1	0.898
Hypoxanthine	9.9	2.3^a^	335.0	36.9^a^	2,212.6	203.3^b^	<0.001
Ile	177.8	17.9^a^	231.0	10.7^a^	512.3	74.8^b^	0.016
IMP	78.4	39.2^a^	7,574.3	1,402.1^c^	3,437.7	205.6^b^	0.006
Inosine	4.5	2.5^a^	543.5	91.7^b^	1,109.1	239.5^c^	0.008
Lactic acid	43,827.0	6,652.1^a^	115,982.1	15,570.4^b^	105,428.9	6,388.3^b^	0.020
Leu	263.3	25.7^a^	314.7	18.7^a^	826.8	132.6^b^	0.019
Lys	341.3	36.0^a^	454.2	54.4^a^	732.6	12.1^b^	0.006
Malic acid	802.1	138.4^b^	329.4	124.6^a^	105.1	28.9^a^	0.017
Met	44.0	4.1^a^	42.6	2.4^a^	323.7	71.0^b^	0.014
NAD+	550.5	59.7^c^	277.9	84.7^b^	20.2	5.2^a^	0.004
Ornithine	84.6	4.8	91.2	8.6	148.5	38.1	0.196
Phe	113.2	10.8^a^	138.2	7.8^a^	419.5	76.2^b^	0.020
Pro	267.4	17.8	275.0	35.1	338.4	26.1	0.212
PRPP	24.0	0.8^b^	0	-a	0	-a	<0.001
Putrescine	9.9	0.9^a^	19.5	0.9^b^	19.3	2.4^b^	0.002
Pyruvic acid	0	-	182.2	182.2	0	-	NA
Ribose 5-phosphate	0	-a	56.6	8.6^b^	70.0	16.6^b^	0.031
Ribulose 5-phosphate	34.9	8.9^a^	180.1	6.2^b^	215.0	48.6^b^	0.035
S-Adenosylmethionine	8.5	2.3^a^	18.6	2.7^b^	4.0	1.3^a^	0.008
Sedoheptulose 7-phosphate	0	-a	29.9	3.1^a^	308.9	48.4^b^	0.002
Ser	290.0	17.1^a^	311.8	19.9^a^	735.1	94.4^b^	0.012
Spermidine	1.6	0.1^a^	22.8	3.3^b^	20.6	2.8^b^	0.002
Spermine	0	-	71.8	22.6	31.0	13.3	0.078
Succinic acid	1,250.1	374.2	1,071.2	180.6	1,953.8	360.0	0.280
Thr	202.5	9.8^a^	219.5	26.4^a^	425.9	41.5^b^	0.018
Trp	38.5	3.8^a^	45.0	4.9^a^	84.7	10.1^b^	0.030
Tyr	89.8	8.2^a^	105.7	3.7^a^	348.3	73.4^b^	0.027
UMP	0.0	-a	85.6	12.6^b^	34.7	7.2^a^	0.003
Uridine	14.1	2.3^a^	29.6	4.6^a^	131.9	11.0^b^	<0.001
UTP	124.5	10.3^b^	0	-a	0	-a	<0.001
Val	362.7	31.3^a^	402.8	28.9^a^	760.3	114.8^b^	0.050
β-Ala	119.8	34.9	94.8	10.9	105.5	22.1	0.849

ANOVA, analysis of variance; SE, standard error; ADP, adenosine diphosphate; AMP, adenosine monophosphate; ATP, adenosine triphosphate; CTP, cytidine triphosphate; GABA, γ-aminobutyric acid; GMP, guanosine monophosphate; GTP, guanosine triphosphate; IMP, inosine 5′-monophosphate; PRPP, phosphoribosyl pyrophosphate; UMP, uridine monophosphate; UTP, uridine triphosphate.

The compounds are quantified by CE-TOFMS and shown in nmol/g. The contents of compounds under detection are indicated as 0. Compounds that were not detected throughout the experimental duration are not listed.

a–c Values with different subscript differ between aging time points (p<0.05).

NA, not applicable due to too large variance between the samples.

**Table 2:** Loading values of top 20 compounds for principal component-1 and -2 of Japanese black beef aging analysis1)

PC1	Compound name	PC2	Compound name
	
0.1184	Cysteine glutathione disulfide	0.1504	N5-Ethylglutamine
0.1177	Galactosamine/Glucosamine	0.1461	S-Methylcysteine
0.1159	Glucose 6-phosphate	0.1427	Malonylcarnitine
0.1157	Nicotinamide	0.1414	Gly
0.1156	Thiamine	0.1384	Terephthalic acid
0.1155	S-Adenosylhomocysteine	0.1344	Urea
0.1153	Cys	0.1330	Betaine
0.1152	Hypoxanthine	0.1302	Glutathione
0.1147	Uridine	0.1269	Methylhistidine
0.1146	Gluconic acid	0.1224	NADH
−0.0947	S-Lactoylglutathione	−0.0766	GMP
−0.0970	Fructose 1,6-diphosphate	−0.0814	Uric acid
−0.1000	Glycerol 3-phosphate	−0.0861	ADP-ribose
−0.1008	UDP-N-acetylgalactosamine/UDP-N-acetylglucosamine	0.0984	Spermine
−0.1010	Malic acid	−0.1018	Nicotinamide mononucleotide
−0.1041	UDP-glucose/UDP-galactose	0.1047	UMP
−0.1041	Citric acid	−0.1095	IMP
−0.1042	Argininosuccinic acid	−0.1209	Pyruvic acid
−0.1111	Trimethylamine N-oxide	−0.1300	AMP
−0.1121	NAD+	−0.1500	IDP

NADH, nicotinamide adenine dinucleotide; GMP, guanosine monophosphate; ADP, adenosine diphosphate; UDP, uridine diphosphate; UMP, uridine monophosphate; IMP, inosine 5′-monophosphate; AMP, adenosine monophosphate; IDP, inosine 5′-diphosphate.

1) Compounds with top 20 positive and negative loading values for PC1 and PC2 are listed.

**Table 3:** Representative KEGG metabolic pathways that contain significantly changed metabolites during postmortem aging of Japanese Black *Longissimus thoracis* muscle1)

KEGG pathway, compounds	Relative content2)						Adjusted p value	Numbers of metabolites

	D0		D1		D14			
		
	Mean	SE	Mean	SE	Mean	SE		
Protein digestion and absorption								16/31
Asn	146.6	11.6	125.9	25.0	234.5	18.8	0.0092	
Asp	62.8	6.2	10.5	8.5	49.8	3.2	0.0084	
Cys	0.6	0.3	29.3	16.9	120.2	69.4	0.0009	
Cystine	0	-	0	-	2.2	0.3	<0.0001	
Glu	444.0	53.4	201.3	63.6	971.5	168.3	0.0067	
His	251.0	10.6	208.7	35.4	368.2	27.1	0.0098	
Ile	715.3	81.9	770.9	151.8	1,757.5	233.9	0.0033	
Leu	1,229.4	52.1	1,234.5	231.2	3,354.1	483.3	0.0019	
Lys	502.3	73.5	524.1	67.4	905.9	30.8	0.0023	
Met	91.9	3.0	79.4	22.7	587.9	113.4	0.0010	
Phe	349.8	17.2	358.7	67.7	1,124.8	180.6	0.0018	
Ser	399.9	30.6	363.3	77.5	872.2	107.5	0.0029	
Thr	369.2	28.5	325.5	52.7	666.3	64.9	0.0032	
Trp	80.4	6.0	76.1	10.7	152.3	16.2	0.0028	
Tyr	182.2	6.2	182.5	37.9	614.9	114.6	0.0032	
Val	1,088.8	45.9	1,005.1	174.8	1,980.0	272.2	0.0090	
(Dipeptides)								6/7
Ala-Ala	0	-	0	-	20.1	4.2	0.0006	
Glu-Glu	0	-	13.1	8.2	75.6	14.2	0.0012	
Gly-Gly	0	-	5.6	2.3	13.7	0.3	0.0031	
His-Glu	0	-	0	-	5.0	1.6	0.0047	
Thr-Asp Ser-Glu	0	-	0	-	10.0	1.8	0.0002	
Tyr-Glu	0	-	2.7	1.7	10.6	1.8	0.0028	
Glycolytic pathway								3/12
Dihydroxyacetone phosphate	245.1	16.0	0	-	0	-	<0.0001	
Fructose 1,6-diphosphate	112.9	11.0	6.7	2.8	0	-	0.0001	
Lactic acid	14,121.1	1,212.1	30,112.3	3571.4	29,663.1	2222.0	0.0173	
Citric acid cycle								3/4
Citric acid	91.8	9.9	13.7	5.9	0	-	0.0004	
Fumaric acid	42.9	6.0	5.1	4.2	17.2	8.4	0.0369	
Malic acid	563.4	39.4	182.8	41.3	65.6	15.8	0.0004	
Pyruvate metabolism								3/9
Dihydroxyacetone phosphate	245.1	16.0	0	-	0	-	<0.0001	
Lactic acid	14,121.1	1,212.1	30,112.3	3571.4	29,663.1	2222.0	0.0173	
Malic acid	563.4	39.4	182.8	41.3	65.6	15.8	0.0004	
Pentose phosphate pathway								4/9
6-Phosphogluconic acid	0	-	1.5	1.2	28.0	5.9	0.0008	
Ribose 5-phosphate	0	-	22.6	5.2	29.0	6.2	0.0252	
Ribulose 5-phosphate	15.0	2.2	68.2	14.4	83.8	17.1	0.0491	
Sedoheptulose 7-phosphate	0	-	11.8	2.0	127.4	14.8	0.0000	
Nicotinate and nicotinamide metabolism								4/15
Dihydroxyacetone phosphate	245.1	16.0	0	-	0	-	<0.0001	
Fumaric acid	42.9	6.0	5.1	4.2	17.2	8.4	0.0369	
NAD+	221.4	13.8	80.9	11.8	4.9	2.3	0.0001	
Nicotinamide	156.8	10.6	386.5	76.3	754.0	73.9	0.0025	
Glycerophospholipid metabolism								4/10
CDP-choline	1.3	1.1	7.4	1.8	8.5	0.4	0.0003	
Choline	94.1	6.7	122.5	20.8	301.9	27.9	0.0314	
Dihydroxyacetone phosphate	245.1	16.0	0	-	0	-	<0.0001	
Glycerol 3-phosphate	1,560.5	193.1	348.0	109.7	30.2	6.2	0.0010	
Purine metabolism								15/50
AMP	0	-	43.3	6.8	25.6	1.6	0.0025	
ADP-ribose	4.6	3.0	9.9	2.2	0	-	0.0057	
ADP	36.8	4.0	17.9	1.8	18.0	2.3	0.0133	
ATP	4,888.2	430.3	11.1	1.0	6.8	2.9	0.0001	
GMP	0	-	43.6	7.4	30.3	2.1	0.0031	
GTP	85.2	11.7	0	-	0	-	0.0005	
Guanosine	0	-	7.7	2.7	21.4	4.3	0.0059	
Hypoxanthine	12.4	1.5	396.9	131.0	2,466.0	186.9	<0.0001	
IMP	26.3	9.5	2,035.0	155.8	1,031.6	64.7	0.0001	
Inosine	3.9	2.5	742.3	265.3	1,407.6	276.1	0.0244	
PRPP	11.3	1.0	0	-	0	-	<0.0001	
Ribose 5-phosphate	0	-	22.6	5.2	29.0	6.2	0.0252	
Uric acid	8.1	1.2	19.0	1.7	10.7	0.4	0.0052	
Xanthine	0	-	57.1	12.4	219.4	22.2	0.0001	
Xanthosine	0	-	2.9	1.4	6.6	0.2	0.0076	
Cysteine metabolism								5/18
Cys	0.6	0.3	29.3	16.9	120.2	69.4	0.0009	
Cystine	0	-	0	-	2.2	0.3	<0.0001	
Glutathione (GSSG)_divalent	192.9	12.0	73.5	8.2	81.0	11.3	0.0011	
Homocysteine	2.8	1.4	4.6	1.9	14.6	0.6	0.0009	
Ser	399.9	30.6	363.3	77.5	872.2	107.5	0.0029	
Glutathione metabolism								4/16
5-Oxoproline	7.8	3.2	21.7	7.2	67.7	12.0	0.0026	
Cys	0.6	0.3	29.3	16.9	120.2	69.4	0.0009	
Glu	444.0	53.4	201.3	63.6	971.5	168.3	0.0067	
Spermidine	2.8	0.1	31.9	3.3	31.3	3.7	0.0014	

KEGG, Kyoto encyclopedia of genes and genomes; SE, standard error; AMP, adenosine monophosphate; ADP, adenosine diphosphate; ATP, adenosine triphosphate; GMP, guanosine monophosphate; GTP, guanosine triphosphate; IMP, inosine 5′-monophosphate; PRPP, phosphoribosyl pyrophosphate.

1) Compounds were extracted and listed if their changes were significant during postmortem aging of Japanese Black cattle *Longissimus thoracis muscle* (adjusted p<0.05, corresponding to false discovery rate<0.1). The relative value of each compound to 3MH is indicated.

2) Numbers of significantly changed compounds/total numbers of the analyzed compounds that belong to the KEGG pathway.
