# Corrigendum

**DOI:** 10.5713/ajas.18.0648C

**Published:** 2019-08-23

The followings are to be changed in Table 2 of **Asian-Australas J Anim Sci 2019. Vol 32, No. 8 : 1172–1185**: PC2 value of spermine and UMP should be −0.0984 and −0.1047, respectively.

