# Supplementary material for: Metabolomic approach to key metabolites characterizing postmortem aged loin muscle of Japanese Black (Wagyu) cattle
Source: Asian-Australas J Anim Sci. 2019 Jan 4;32(8):1172–85. doi: 10.5713/ajas.18.0648 (PMC6599950; doi:10.5713/ajas.18.0648)
Supplement: Supplementary file 1 [file ajas-18-0648-suppl.pdf]

Supplemental Table 1. The top 50 compounds that showed significant increase or decrease during the postmortem aging

metabolite	D0_1	D0_2	D0_3	D1_1	D1_2	D1_3	D14_1	D14_2	D14_3	p_masigpro	rank_masigpro
N-Acetylglucosamine 6-phosphate	-0.75	-0.75	-0.75	-0.75	-0.17	-0.75	1.2	1.3	1.4	1.59E-06	1
Hypoxanthine	-0.81	-0.81	-0.81	-0.21	-0.6	-0.64	1.7	0.98	1.2	1.08E-05	2
Trimethylamine	-0.65	-0.65	-0.65	-0.65	-0.65	-0.65	1.3	0.78	1.7	1.17E-05	3
Dihydroxyacetone phosphate	1.3	1	1.6	-0.66	-0.66	-0.66	-0.66	-0.66	-0.66	1.20E-05	4
S-Lactoylglutathione	1.6	0.99	1.3	-0.66	-0.66	-0.66	-0.66	-0.66	-0.66	1.34E-05	5
Sedoheptulose 7-phosphate	-0.74	-0.74	-0.74	-0.47	-0.59	-0.59	0.72	1.6	1.5	1.62E-05	6
Cystine	-0.64	-0.64	-0.64	-0.64	-0.64	-0.64	1.2	1.9	0.84	2.82E-05	7
UTP	1.6	0.91	1.4	-0.66	-0.66	-0.66	-0.66	-0.66	-0.66	3.17E-05	8
Phosphoribosyl diphosphate	1.7	1	1.2	-0.65	-0.65	-0.65	-0.65	-0.65	-0.65	3.61E-05	9
ATP	1.7	0.93	1.3	-0.66	-0.65	-0.65	-0.66	-0.66	-0.65	5.07E-05	10
Uridine	-0.77	-0.83	-0.72	0.018	-0.71	-0.78	1.7	1	1.1	6.35E-05	11
NAD+	1.4	0.88	1.4	-0.42	0.071	-0.32	-1	-0.96	-1.1	6.46E-05	12
Fructose 1,6-diphosphate	1.1	1.1	1.8	-0.71	-0.53	-0.53	-0.71	-0.71	-0.71	7.56E-05	13
Thiamine	-0.91	-0.91	-0.91	-0.12	-0.41	-0.48	1.4	0.75	1.6	7.69E-05	14
IMP	-1.1	-1.1	-1.1	1.4	1.3	0.7	-0.065	0.18	-0.11	7.90E-05	15
Xanthine	-0.9	-0.9	-0.9	-0.045	-0.49	-0.49	0.82	1.2	1.7	9.34E-05	16
Thr-Asp<br>Ser-Glu	-0.62	-0.62	-0.62	-0.62	-0.62	-0.62	2	0.73	0.97	0.000167	17
Cysteine glutathione disulfide	-0.99	-0.99	-0.99	0.21	-0.17	-0.73	1.3	1.2	1.2	0.000175	18
CTP	1.9	1.1	0.97	-0.65	-0.65	-0.65	-0.65	-0.65	-0.65	0.000229	19
Choline	-0.6	-0.83	-0.83	-0.28	-0.2	-0.97	0.58	1.6	1.5	0.00026	20
UDP-glucose<br>UDP-galactose	1.4	1.2	1.2	-0.78	-0.78	0.074	-0.78	-0.78	-0.78	0.000275	21
Malate	1	1.1	1.7	-0.12	-0.81	-0.2	-0.71	-0.97	-0.95	0.000425	22
Citrate	1.7	0.77	1.3	-0.79	-0.41	-0.24	-0.79	-0.79	-0.79	0.000426	23
GTP	1.9	0.78	1.2	-0.64	-0.64	-0.64	-0.64	-0.64	-0.64	0.000491	24
Gluconate	-0.95	-0.95	-0.95	-0.082	-0.38	-0.33	1.6	1.5	0.6	0.000528	25
Ala-Ala	-0.61	-0.61	-0.61	-0.61	-0.61	-0.61	2.2	0.73	0.79	0.00061	26

Trimethylamine N-oxide	0.92	0.36	1.6	0.35	0.14	0.28	-1.3	-0.94	-1.3	0.00064	27
Gluconolactone	-1	-1	-1	0.024	0.15	-0.48	1.1	1.7	0.7	0.000804	28
6-Phosphogluconic acid	-0.65	-0.65	-0.65	-0.65	-0.65	-0.36	1.2	2.1	0.4	0.000815	29
Cys	-0.85	-0.82	-0.85	0.082	-0.4	-0.73	2	0.84	0.74	0.000886	30
Homocysteine	-0.2	-0.82	-1.2	-0.17	0.017	-1.2	1.2	1.4	0.97	0.00091	31
Glycerol 3-phosphate	1.4	0.63	1.7	-0.33	-0.13	-0.75	-0.85	-0.81	-0.84	0.001032	32
Met	-0.55	-0.6	-0.58	-0.42	-0.7	-0.74	2.2	0.57	0.85	0.001049	33
GSSG	1.5	0.78	1.5	-0.39	-0.95	-0.73	-0.89	-0.69	-0.13	0.001066	34
Glu-Glu	-0.76	-0.76	-0.76	0.093	-0.64	-0.72	2	0.52	0.99	0.001201	35
Spermidine	-1.2	-1.3	-1.3	1.1	0.64	0.19	0.14	1.1	0.53	0.00137	36
UDP-N-acetylgalactosamine UDP-N-acetylglucosamine	1.2	0.61	1.9	-0.19	-0.39	-0.51	-0.94	-0.89	-0.75	0.001394	37
Phe	-0.53	-0.69	-0.57	-0.21	-0.67	-0.85	2.2	0.52	0.83	0.001763	38
N-Acetyllysine	-0.71	-0.71	-0.71	-0.075	-0.58	-0.71	2.2	0.68	0.64	0.001783	39
Leu	-0.52	-0.69	-0.56	-0.13	-0.69	-0.93	2.2	0.59	0.78	0.001918	40
Galactosamine Glucosamine	-1.1	-1.1	-1.1	0.53	0.13	-0.53	1.5	0.55	1.1	0.002145	41
Lys	0.018	-1.4	-0.55	0.13	-0.59	-1.1	1.4	1.2	0.86	0.002259	42
Dyphylline	-1	-1.5	-0.98	0.87	0.026	-0.27	1.1	1.2	0.62	0.002299	43
Nicotinamide	-1.1	-0.94	-0.91	0.46	-0.66	-0.28	1.7	1.1	0.59	0.002489	44
AMP	-1.1	-1.1	-1.1	0.73	0.47	1.8	0.32	-0.004	0.065	0.002521	45
5-Oxoproline	-1	-0.7	-0.63	0.22	-0.59	-0.67	0.45	0.92	2	0.002628	46
Cytidine	-1.1	-0.83	-0.67	0.64	-0.65	-0.77	1.3	0.66	1.4	0.002638	47
Trp	-0.32	-0.86	-0.4	-0.068	-0.68	-1.1	2.1	0.53	0.87	0.002798	48
Tyr-Glu	-0.82	-0.82	-0.82	0.42	-0.55	-0.82	1.9	0.91	0.55	0.002849	49
Ser	-0.25	-0.6	-0.68	-0.013	-0.75	-1.1	2.1	0.85	0.54	0.002949	50
